# Endocrine Disrupting Chemicals and Thyroid Cancer: An Overview

**DOI:** 10.3390/toxics9010014

**Published:** 2021-01-19

**Authors:** Mathilda Alsen, Catherine Sinclair, Peter Cooke, Kimia Ziadkhanpour, Eric Genden, Maaike van Gerwen

**Affiliations:** 1 Department of Otolaryngology-Head and Neck Surgery, Icahn School of Medicine at Mount Sinai, New York, NY 10029, USA; mathilda.alsen@mountsinai.org (M.A.); Catherine.Sinclair@mountsinai.org (C.S.); Eric.Genden@mountsinai.org (E.G.); 2 Department of Medical Education, Icahn School of Medicine at Mount Sinai, New York, NY 10029, USA; peter.cooke@icahn.mssm.edu (P.C.); kimia.ziadkhanpour@icahn.mssm.edu (K.Z.); 3 Institute for Translational Epidemiology, Icahn School of Medicine at Mount Sinai, New York, NY 10029, USA

**Keywords:** endocrine disruptive chemicals, thyroid cancer, pesticides, flame retardants, polychlorinated biphenyls, phthalates, perfluoroalkyl substances, bisphenol A

## Abstract

Endocrine disruptive chemicals (EDC) are known to alter thyroid function and have been associated with increased risk of certain cancers. The present study aims to provide a comprehensive overview of available studies on the association between EDC exposure and thyroid cancer. Relevant studies were identified via a literature search in the National Library of Medicine and National Institutes of Health PubMed as well as a review of reference lists of all retrieved articles and of previously published relevant reviews. Overall, the current literature suggests that exposure to certain congeners of flame retardants, polychlorinated biphenyls (PCBs), and phthalates as well as certain pesticides may potentially be associated with an increased risk of thyroid cancer. However, future research is urgently needed to evaluate the different EDCs and their potential carcinogenic effect on the thyroid gland in humans as most EDCs have been studied sporadically and results are not consistent.

## 1. Introduction

The incidence of thyroid cancer has been steadily increasing in recent decades in the United States and worldwide [1]. Although early or incidental detection of smaller tumors due to more advanced and frequent use of imaging technology may partially explain this increase [2], research has highlighted the potential contribution of exposure to environmental pollutants to this phenomenon [3]. Several studies have suggested that exposure to certain endocrine disruptive chemicals (EDCs) alter thyroid function and is associated with increased risk of numerous adverse health outcomes including developmental abnormalities, thyroid disorders and various types of cancer [4,5,6,7]. In 2002, the World Health Organization (WHO) and the International Programme on Chemical Safety (IPCS) defined an EDC as “an exogenous substance or mixture that alters function(s) of the endocrine system and consequently causes adverse health effects in an intact organism, or its progeny or (sub-) populations” [8]. Known and suspected EDCs include pesticides, flame retardants, polychlorinated biphenyls (PCBs), phthalates, perfluoroalkyl substances (PFAS) and Bisphenol A (BPA) [9,10]. The present paper aims to provide a comprehensive overview of thyroid cancer risk associated with exposure to EDCs.

## 2. Materials and Methods

Original studies evaluating thyroid cancer risk associated with EDC exposure were identified in the National Library of Medicine and National Institutes of Health PubMed through November 2020. Although many environmental chemicals may have endocrine disrupting activity, the current study focused on the most commonly studied EDCs and grouped them as followed: (1) Industrial: flame retardants (polybrominated biphenyls (PBB), polybrominated diphenyl ethers (PBDEs), organophosphate flame retardants (PFRs)), PCBs, (2) Plastics/plasticizers: phthalates, BPA, (3) PFAS, (4) Agricultural: organochlorine pesticides, organophosphate pesticides, and other pesticides. To identify relevant publications, the following keyword search terms were used in different combinations depending on the EDC that was researched: “thyroid cancer”, “endocrine disrupting chemicals” or “endocrine disruptors”, “flame retardants”, “polybrominated biphenyls” or “PBB”, “polybrominated diphenyl ethers” or “PBDEs”, “organophosphate flame retardants”, “polychlorinated biphenyls” or “PCBs”, “phthalates”, “bisphenol A” or “BPA”, “perfluoroalkyl acids” or “PFAS”, “perfluorooctanoic acid” or “PFOA”, “perfluorooctane sulfonate” or “PFOS”, “pesticides”, “organochlorine pesticides”, “organophosphate pesticides”, “insecticides”, “organochlorine insecticides”, “organophosphate insecticides”, “herbicides”, “organochlorine herbicides”, “organophosphate herbicides”, “carbamates”, “fungicides”. Reference lists of all retrieved articles and of previously published relevant reviews were also reviewed for additional studies. One investigator performed the literature search (M.A.) and consulted with a second investigator (M.v.G.) to discuss eligibility for inclusion. Eligible studies for inclusion were case-control studies, cohort studies, and cross-sectional studies. Reviews, systematic reviews and meta-analyses were excluded but reviewed to retrieve additional eligible individual studies. Letters, animal studies, commentaries and in vitro/in vivo studies were excluded. From the eligible studies, we obtained adjusted odds ratios (OR), hazard ratios (HR) or risk ratios (RR) and 95% confidence intervals (CI). All reported OR, HR, or RR are adjusted unless otherwise specified. Certain studies reported standardized incidence ratios (SIR) or standardized mortality ratios (SMR). We identified nine eligible studies on thyroid cancer and industrial EDCs, five studies on thyroid cancer and plastics/plasticizers, four studies on thyroid cancer and PFAS and fourteen studies on thyroid cancer and EDCs in agriculture.

## 3. Results

### 3.1. Industrial: Flame Retardants

The use of flame retardants has increased significantly over the last several decades with the purpose of reducing flammability of products [11]. Flame retardants can be found in various household and commercial products including electronic devices, textiles, furniture, building insulation and children’s toys [11,12,13]. Major routes of exposure are through skin contact, diet, inhalation and ingestion of indoor air and settled dust [14,15]. In the 1970s, polybrominated biphenyls (PBB) and polybrominated diphenyl ethers (PBDEs), which have similar chemical structures, served as brominated flame retardants in furniture, electrical equipment and other household products [16]. The usage of PBBs was banned in the early 1970s after the toxin was accidentally mixed into meat, egg and dairy products, exposing millions of people to contaminated food [16,17]. Following the ban, PBDEs replaced PBBs as flame retardants. More recently, some PBDEs have also been either banned or reduced in production due to growing health concerns, although the exact human health effects are still unknown [18]. Despite the phase out in production, PBDEs are still detected in the environment and in household dust [19]. In addition, PBDEs have an estimated half-life of 1–12 years in humans [20], whereas PBBs have an estimated half-life of 10.8 years [21].

Previous studies have linked flame retardants to various adverse health outcomes such as diabetes [22], neurobehavioral disorders [23] and reproductive health effects [24]. Flame retardants have also been shown to alter thyroid hormone action [25]. A study including papillary thyroid cancer (PTC) patients showed that hydroxylated PBDEs were negatively associated with free thyroxine (FT4) but positively associated with thyroid-stimulating hormone (TSH), suggesting that OH-PBDEs alter thyroid function in PTC patients [26].

PBBs and PBDEs have similar proposed carcinogenic mechanisms both via the creation of DNA adducts and disruptions in thyroid homeostasis, due to similar chemical structures. Hydroxylated PDBEs and PBBs competitively bind thyroid-associated proteins thus displacing thyroxine (T4) and lowering the hormone’s half-life [27]. Additionally, PBDEs and PBBs lower the half-life of thyroid hormone through the induction of UDP glycosyltransferase in the liver, which in turn leads to increased glucuronidation of T4 and subsequent biliary excretion [28,29]. These disruptions in thyroid hormone metabolism may contribute to dysregulated cell proliferation [30]. Furthermore, PBDE quinones have been shown to bind DNA adducts with potential carcinogenic effects. The International Agency for Research on Cancer (IARC) has classified PBBs as probably carcinogenic to humans (group 2A) [31]. The United States (US) Department of Health and Human Services similarly regards PBBs as reasonably carcinogenic based on sufficient evidence from experimental studies [32]. IARC stated that PBDEs are not classifiable by their carcinogenicity in humans, based on inadequate evidence from experimental studies. Nevertheless, the Environmental Protection Agency (EPA) suggests that there is evidence of carcinogenic potential for decaBDE, a mixture of different PBDE congeners [33].

Some studies have suggested a positive association between flame retardants and thyroid cancer (Table 1). Hoffman et al. examined dust and serum samples in patients diagnosed with PTC and demonstrated that higher levels of some classes of flame retardants were associated with an increased risk of PTC [34]. Patients with a high exposure (above the median value) to decabromodiphenyl ether (BDE-209), a PBDE congener, or tris(2-chloroethyl) phosphate (TCEP) were more likely to be diagnosed with PTC compared to patients with low (below the median value) exposure, OR: 2.29 (95% CI: 1.02–5.08) and OR: 2.42 (95% CI: 1.10–5.33), respectively (Table 1). The association with BDE-209 was particularly strong in patients with tumor stage 1a or 1b PTC (OR: 3.22 (95% CI: 1.16–2.11), while the association with TCEP was stronger in patients with tumor stage 2, 3, or 4 PTC (OR: 3.18 (95% CI: 1.08–9.38) [34].

Huang et al. examined the association between PTC and seven PBDE congeners and one PBB congener among US military personnel [35]. Participants with a high serum BDE-28 level (highest tertile) had an increased risk of PTC (OR: 2.09; 95% CI: 1.05–4.15), compared to serum levels below the limit of detection; the association was particularly strong in tumors measuring >10 mm (OR: 4.77; 95% CI: 1.84–12.35) and in females (OR: 10.74; 95% CI: 1.93–59.72) [35].

Other studies have shown no association between flame retardants and thyroid cancer (Table 1). Aschebrook et al. evaluated blood levels of PBDEs and the risk of thyroid cancer using a nested case control study within the prostate, colorectal, lung and ovarian cancer screening trial cohort, but found no association between exposure to PBDE and thyroid cancer [36]. Deziel et al. (2019) conducted a case-control study examining the association of PBDEs/PBBs serum levels and the risk of PTC among women in Connecticut and found no evidence of an increased risk of thyroid cancer, however there was some evidence of an adverse association [37].

Alternate flame retardants, such as organophosphate flame retardants (PFRs), which are currently used in larger volumes, also have shown to alter thyroid function [38]. To date, only one known study has looked at the association between organophosphate flame retardants and thyroid cancer. Deziel et al. (2018) conducted a case-control study to explore this association in women and reported no association between PFRs and thyroid cancer [39].

In summary, only few studies have assessed the association between flame retardants and thyroid cancer and reported conflicting results, potentially due to different congeners and different routes of exposure.

### 3.2. Industrial: Polychlorinated Biphenyls (PCBs)

Polychlorinated Biphenyls (PCBs) are a group of organic chemicals that were previously used in commercial and industrial production of electronic devices, plasticizers, pigments and flame retardants as well as cooling agents in electronic transformers and capacitors [40,41]. PCBs were banned in 1979 due to the high toxicity and suggested threats to human health and wildlife [42]. Despite the ban, PCB residues remain in the environment due to their persistence and bioaccumulation and can still be found in products including electrical equipment, oil, cable, insulation, plastics and paint [43]. The estimated half-life of PCBs is 10–15 years [44].

Exposure to PCB’s has been associated with increased cancer risk. A meta-analysis including 16 studies found a significant association between PCB exposure and breast cancer among those with higher plasma levels of the following PCB congeners: PCB-99 (OR: 1.36; 95% CI: 1.02–1.80), PCB-183 (OR: 1.56; 95% CI: 1.25–1.95) and PCB-187 (OR: 1.18; 95% CI: 1.01–1.39) [45]. There are two proposed mechanisms for PCB carcinogenesis in the thyroid, namely the activation of a key oncogenic transcription factor and the multifaceted disruption of thyroid homeostasis. First, dioxin-like PCBs agonistically bind the aryl hydrocarbon receptor (AHR), a transcription factor involved in many aspects of tumorigenesis, including initiation progression and metastasis [46]. Cytoplasmic ligand binding of the AHR leads to translocation of the transcription factor into the nucleus and subsequent transcription of genes including CYP1A1, a known hepatic metabolizer of multiple pro-carcinogens [47]. Secondly, researchers have proposed that PCBs exert their carcinogenic effects via altering thyroid homeostasis. Some studies provided evidence that PCBs directly disrupt thyroid hormone synthesis although the results have been inconsistent [48,49,50,51,52]. Additionally, hydroxylated PCBs (OH-PCBs) have structural similarities to thyroid hormones triiodothyronine (T3) and T4. These OH-PCBs bind thyroid associated proteins, such as transthyretin and thyroxin-binding globulin, thus disrupting normal hypothalamic-pituitary-thyroid axis pathways and feedback loops [53]. Research into the association between PCBs and any type of cancer has shown conflicting results, although PCBs have shown to cause cancer in animals [43]. The EPA has therefore determined that PCBs are probable human carcinogens [33]. IARC has similarly classified PCBs a group 1 carcinogen, meaning there is enough evidence to conclude that PCBs can cause cancer in humans [31].

Studies exploring the association between PCB exposure and thyroid cancer are limited (Table 2). Zhuo et al. found a significant dose-response relationship between PCB-118 congener and an increased risk of thyroid cancer in 1484 participants recruited from the US military [54]. A lipid-adjusted PCB-118 serum concentration of 6.61–43.68 ng/g (4th quartile) was significantly associated with a higher risk of thyroid cancer compared to a lipid-adjusted serum concentration below the limit of detection (LOD: 2.42 ng/g; 1st quartile) (OR: 1.55; 95% CI: 1.01–2.38). After stratification by gender, they found a significant positive association between PCB-118 exposure and the risk of thyroid cancer in the 2nd (serum concentration of 2.43–4.10 ng/g) vs. 1st quartile in females (OR: 1.90; 95% CI: 1.10–3.27) and males (OR: 1.96; 95% CI: 1.16–3.33) [54]. In a nested case-control study by Lerro et al. (2018) PCBs were not associated with thyroid cancer with the exception of congener PCB-114 which was present at low concentrations and inversely associated with thyroid cancer (OR per 1 ng/g increase: 0.78; 95% CI: 0.62–0.97) [55]. The same study found a positive association between total PCB exposure (OR: 1.25; 95% CI: 1.00–1.56), PCB 138/158 (OR: 4.54; 95% CI: 1.20–17.2) and PCB 153 (OR: 3.47; 95% CI: 1.18–10.2) with thyroid cancer but only among individuals in the youngest birth cohort (individuals born between 1943–1957) [55]. Similarly, Deziel et al. (2020) found no significant association between PCBs and PTC among 250 female incident cases and 250 controls in Connecticut. However, there was a significant association between PCB 74 (OR: 2.22 95% CI; 1.05–4.72), PCB 114 (OR: 2.03; 95% CI:1.04–3.97), PCB 146 (OR: 2.27; 95% CI: 1.15–4.47), PCB 153 (OR: 2.19; 95% CI: 1.11–4.33), PCB 156 (OR: 2.58; 95% CI: 1.22–5.50), PCB 157 (OR: 2.39; 95% CI: 1.16–4.91), PCB 167 (OR: 2.15; 95% CI: 1.11–4.20), PCB 178 (OR: 2.02; 95% CI: 1.03–3.93), PCB 187 (OR: 1.96; 95% CI: 1.02–3.75) and the risk of PTC among those who were born after the year 1960 when there was a peak production in PCBs [56]. The studies by Lerro et al. (2018) and Deziel et al. (2020) potentially suggest that PCB exposure during childhood may increase the risk of thyroid cancer later in life. A cohort study by Ruder et al. of 24,864 workers previously exposed to PCBs found no association between PCBs and thyroid cancer mortality for the entire cohort (standardized mortality ratio (SMR): 0.52; 95% CI: 0.11–1.53), short-term workers employed less than 3 months (SMR: 0.00; 95% CI 0.00–2.76), and long-term workers employed 3 months or longer (SMR: 0.68; 95% CI: 0.14–2.00) [57].

Based on existing studies exploring the relationship between PCB exposure and thyroid cancer, the evidence of an association is limited, although the exposure of PCBs at a young age should be further investigated.

### 3.3. Plastic and Plasticizers: Phthalates

Phthalates are used as plasticizers in different products including cosmetics, shampoos, soaps, toys, detergents, food packaging, medical products and pharmaceuticals [58]. It is currently unclear how phthalates may affect human health, although an association between phthalate exposure with various adverse health outcomes in humans including asthma [59,60], breast cancer [61] and male reproductive issues [62], has been reported. Phthalates have been shown to disrupt endocrine systems and affect thyroid hormone homeostasis and growth of thyroid [63,64,65] An extensive meta-analysis evaluating urinary concentration of di(2-ethylhexyl)phthalate (DEHP) metabolites (mono-ethylhexyl phthalate (MEHP), mono (2-ethyl-5-hydroxyhexyl) phthalate (MEHHP) and mono (2-ethyl-5-oxohexyl) phthalate (MEOHP) and their association with thyroid hormones (TSH, fT4 and TT4) [66], suggested that higher levels of the MEHP/MEHHP metabolites were associated with a decrease in TT4 (pooled correlation coefficients −0.02, 95% CI: −0.05; 0.00 and −0.03, 95% CI: −0.05; −0.01), whereas the MEOHP metabolite was associated with an increase in TSH levels (pooled correlation coefficient 0.02, 95% CI: 0.00; 0.04), indicating that DEHP metabolites may affect thyroid function [63]. However, the half-life of DEHP is relatively short (less than 24 h) [42], and a single measurement only represents current exposure [63].

The proposed potential mechanisms of phthalate carcinogenesis center around the effects on thyroid metabolism and the production of reactive oxygen species (ROS) [66,67,68]. Phthalates are known to mechanistically disrupt thyroid function by inhibiting expression of the sodium-iodide transporter (NIS) by competitively binding the enzyme thyroid peroxidase (TPO) [67]. Studies hypothesized that these disruptions in thyroid homeostasis may play a role in cancer development [66]. In rat models, certain phthalates have been shown to increase ROS in the thyroid [67,68]. In particular, DEHP increases superoxide dismutase activity and thiobarbituric acid reactive substance levels [68]. ROS in thyroid cells have been shown to increase mitogen-activated protein kinase (MAPK) and phosphatidylinositol 3‑kinase (PI3K) pathways, leading to increased cell proliferation [66]. The EPA has classified DEHP as a probable human carcinogen after evidence demonstrating increased liver tumors in rats [33]. IARC has classified DEHP as possibly carcinogenic to humans, although the evidence is far from conclusive [31]. Similarly, DEHP is reasonably anticipated to be human carcinogen, as stated by the Centers for Disease Control and Prevention (CDC) [32] and U.S. Department of Health and Human Services (HHS) [69].

Few studies have found suggestive evidence of a positive association between certain phthalate metabolites and the risk of thyroid cancer (Table 3). Marotta et al. investigated 14 different EDCs detected in serum samples in patients with benign thyroid nodules and patients with differentiated thyroid cancer (DTC), including DEHP and MHEP, and demonstrated that patients with reported serum levels of DEHP had a significantly higher risk of DTC compared to unexposed patients (OR: 15.07; 95% CI: 1.59–142.13) [70]. A case-control study by Miao et al. investigating six urinary phthalate metabolites and thyroid cancer demonstrated that the summation of DEHP metabolites was positively associated with PTC (OR: 3.51; 95% CI: 1.64–7.49). More specifically, the DEHP metabolites associated with an increased risk of PTC were MEHP, MEOHP, mono(2-ethyl-5-carboxypentyl) phthalate (MECPP) and MEHHP. There was no association found between thyroid cancer and monobutyl phthalate (MBP) and Monoethyl phthalate (MEP), which are metabolites of di-n-butyl phthalate (DBP) and diethyl phthalate (DEP), respectively [66]. A case-control study by Liu et al. investigating the association between urinary phthalate metabolites and thyroid cancer showed a significant positive association for mono-methyl phthalate (MMP) (OR: 1.11; 95% CI: 1.01–1.22), MEHHP (OR: 1.53; 95% CI: 1.19–1.96) and MEHP (OR: 1.46; 95% CI: 1.09–1.914) [71]. Urinary MBP and monobenzyl phthalate (MBzP) were inversely associated with thyroid cancer (OR: 0.45; 95% CI: 0.34–0.60) and (OR: 0.71; 95% CI: 0.60–0.85) respectively [71]. There was a difference for gender where the association of MEHP with thyroid cancer was significant in females (OR: 1.90 (95% CI: 1.06–3.41) and non-significant in males (OR: 1.33 (95% CI: 0.95–1.86) [71] (Table 3).

In summary, there are some indications suggesting that phthalate metabolites, including DEHP, may increase the risk of thyroid cancer although certain phthalate metabolites were inversely associated with thyroid cancer risk.

### 3.4. Plastic and Plasticizers: Bisphenol A (BPA)

BPA is used as plasticizer in various products including plastic food containers, water bottles, toys, consumer electronics and medical devices [72,73]. BPA is also used in the production of epoxy resins, which are then used as coating for metal products including food cans, bottle tops and water pipes [72,73]. Studies have shown that BPA from food and drink containers can be transferred to humans and lead to negative health effects including possible reproductive issues in both women and men [74,75]. Research has also demonstrated that most humans have levels of BPA in their urine, serum and plasma as well as in tissue [73,76]. BPA has an estimated half-life of 4–5 h in adult humans [42]. Several thyroid-specific tumorigenic mechanisms of BPA have been outlined in the literature. At the cell membrane level, BPA stimulates the proto-oncogenic estrogen receptor, mER, which triggers downstream activation of the PI3K and MAPK cell proliferation pathways [77]. With regards to BPA cytoplasmic activity, an in vitro study of immortalized thyrocytes (FRTL-5) showed that a very low dose of BPA activates NF-kB, a known transcription factor involved in development of thyroid cancer [78]. Other studies using FRTL cell lines showed that BPA inhibits the expression of DNA damage response enzymes, including Tp53, Af4, E2f5, Smad6 [79]. The downregulation of DNA repair is believed to allow carcinogenic mutations to multiply. BPA has not yet undergone a complete evaluation to determine evidence of human carcinogenic potential [31,32,33]. However, based on a review by Seachrist et al. it was concluded that BPA may be anticipated to be a human carcinogen in the breast and prostate. Authors made this conclusion based on the tumor promoting properties of BPA [80].

Studies investigating the association between BPA and thyroid cancer are limited (Table 4). A case-control study by Zhou et al. showed that urinary BPA levels were significantly higher in patients with PTC (OR: 3.57; 95% CI: 1.37–9.30) compared to healthy controls [81]. Marotta et al. found that serum levels BPAF, an analogue to BPA, was positively associated with differentiated thyroid cancer (OR: 15.07; 95% CI: 1.59–142.13) [70].

### 3.5. Polyfluoroalkyl Substances (PFAS)

PFAS are synthetic chemicals introduced in the 1940s, which can be found in food, household products and drinking water [82]. PFOA and PFOS are no longer manufactured in the US because major American chemical companies voluntarily agreed to eliminate the use of PFOA and PFOA-related chemicals in the early 2000s [82]. However, PFAS can still be found in the environment and various products because of accumulation over time due to their stable chemical structure [42,82,83,84]. PFAS appear to have a biological half-life of up to 8 years [42]. A study by Li et al. determined the mean estimated half-life of PFOA and PFOS to be 2.7 years, and 2.4 years, respectively, in serum samples after exposure to contaminated drinking water [85].

The most studied PFAS compounds are PFOA and PFOS. A potential link between exposure to PFOS and thyroid hormone disruption, including hypothyroidism, has been reported [82,86]. Previous studies suggested that PFAS may exert carcinogenic effects in several ways, primarily through oxidative damage, immunosuppression, and cellular pathway disruption. There is strong evidence indicating that PFAS, particularly the long chain variety (e.g., PFOA), increase ROS [87]. Furthermore, both long-chain and short-chain PFAS exposure result in some degree of immunosuppression. This includes a reduction in natural killer (NK) cell activity and T-cell dependent antibody response [87]. Additionally, PFAS disrupt thyroid hormone physiology by competing at thyroid hormone binding sites and by inducing T4 glucuronidation and its subsequent excretion [87]. Furthermore, some PFAS activate peroxisome proliferator-activated receptors, namely PPARα, a known component in the development of thyroid cancers [88]. Although the EPA classified PFOA as a confirmed animal carcinogen with unknown relevance to humans, the EPA stated that there is suggestive evidence that PFOS and PFOA may cause cancer [33]. IARC has determined PFOA to be a possible carcinogenic to humans [31]. Animal studies have shown that PFOA and PFOS can cause cancer in the liver, testes, pancreas and thyroid [69].

Nevertheless, there are few studies exclusively examining the link between exposure to PFAS and the risk of thyroid cancer (Table 5). Barry et al. investigated the association between exposure to PFOA and cancer risk among 32, 254 participants exposed to contaminated water and found a positive, non-significant association between exposure to PFOA and thyroid cancer (HR: 1.10; 95% CI: 0.95–1.26) [89]. A study by Vieria et al. investigating the association between PFOA exposure and cancer among residents exposed to drinking water contaminated by the DuPont Teflon-manufacturing plant did not find an association between PFOA and thyroid cancer; highest serum PFOA exposure category compared to control cancer cases from the cancer registry (OR: 0.8; 95% CI: 0.2–3.5) [90]. A study by Olsen et al. including 652 participants with occupational exposure to PFOA and 659 controls investigated the risk ratio for thyroid cancer associated with PFOA exposure. No risk ratio could be calculated because no thyroid cancer was diagnosed in the control group while only one was diagnosed in the exposed group (it has to be noted that this study was conducted by the medical department of 3M Medical Company) [91]. Leonard et al. included a cohort of 6027 workers at the DuPont Washington Works plant between 1948 and 2002 and found no difference in thyroid cancer mortality rates between the DuPont Washington Works plant workers and workers at other DuPont sites. However, it must be noted that the number of thyroid-related deaths was very low in the exposed workers group (*n* = 3) and this study was conducted by the DuPont Epidemiology Program [92].

### 3.6. Agricultural Pesticides

Agricultural pesticides are chemicals used to destroy or mitigate pests (insecticides), weeds (herbicides), or fungi (fungicides) [93,94]. Some pesticides are potentially toxic to both animals and humans, and have been linked to negative health effects such as miscarriage and birth defects [94]. Most pesticides contain a combination of active ingredients, exposing workers in agriculture to different toxic chemicals [93,95]. Certain pesticides are known thyroid disrupting chemicals as they imitate thyroid hormones [93] and are potentially a risk factor for cancer, as there have been animal studies showing tumor formation associated with pesticide exposure [96]. Three agricultural regions in Minnesota where pesticides are used were compared to a forested and urban region to examine increased rates of any cancer. Results showed there was an increased mortality rate ratio of thyroid cancer in men (SRR: 2.95; 95% CI: 1.35–6.44) in a region where the pesticide ethylenebisdithiocarbamate was frequently used [97]. Zeng et al. investigated occupational exposure to biocides, a broader category including preservatives, insecticides, disinfectants and pesticides, among workers exposed to these chemicals and found a significant association with an increased risk of thyroid cancer among those ever being occupationally exposed compared to never exposed (OR: 1.65; 95% CI: 1.16–2.35) [98].

Pesticides can be divided by chemical group, of which organochlorine (OC) pesticides and organophosphate (OP) pesticides are among the most common types [99].

### 3.7. Organochlorine Pesticides

OC pesticides consist of a group of synthetic chlorinated hydrocarbon compounds mainly used from the 1940s through the 1960s [93]. Many types of OC pesticides were later banned in the US due to their high toxicity, persistence, lipid solubility, bioaccumulation, and long half-life of 10–15 years [99]. An example is dichlorodiphenyltrichloroethane (DDT) which was banned in 1972 [100]. DDT was developed in the 1940s and mainly used to prevent vector-borne diseases including malaria and typhus [100]. Since the Stockholm Convention (2001), an international environmental treaty aimed at eliminating the production of DDT due to growing health concerns, worldwide DDT use has declined. However, India still reports production and usage of DDT and China only suspended the production of DDT in 2008. Although not all health effects of exposure to OC pesticides are currently known [100], the EPA has determined that evidence from previous findings indicates that OC pesticides as probable human carcinogens after studies showed development of liver tumors in animals exposed to DDT [100]. CDC, HHS and IARC have similarly classified DDT as a possible or probable human carcinogen [31,32,33]. DDT metabolites have been associated with breast cancer in young adult women, indicating that the hormone-disruptive effects of OCs may influence carcinogenesis only during specific developmental periods [101]. Increased risk of aggressive prostate cancer was found associated with OC insecticide. Furthermore, a higher rate of thyroid disease was found among women married to men using pesticides such as aldrin, DDT and lindane compared to women in non-agricultural studies [102]. OC pesticides are both stable and lipophilic, allowing them to remain in tissue for years after initial exposure and potentially exert their tumorigenic effects through different mechanisms [103]. For example, DDT has been shown to compete with thyroid hormone for carrier proteins [103]. Additionally, DDT increases hepatic microsomal enzymes and biliary excretion of T4, leading to a hyperactive thyroid [103]. Other OC pesticides like toxaphene and lindane have been shown to induce chromosomal abnormalities [31,103].

Hexachlorobenzene (HCB), an OC compound formerly used as a pesticide until its ban in 1965, is currently formed as a byproduct during the manufacture of other chemicals [104]. The EPA has classified HCB as a probable human carcinogen, based on animal studies that have reported an increased risk of thyroid, liver and kidney cancer after oral exposure to HCB [33]. IARC has similarly classified HCB as a group 2B carcinogen, meaning possibly carcinogenic to humans [31].

Pentachlorophenol (PCP) is another OC pesticide mainly used to preserve wood, protecting it from fungal rot and insects [105]. The EPA has classified PCPs as likely carcinogenic to humans [33]. IARC has similarly classified PCPs as a group 2B carcinogen, meaning possibly carcinogenic to humans [31].

Lerro et al. (2018) investigated the association between OC pesticide exposure and thyroid cancer in 108 participants and found an inverse association between DDT metabolites and thyroid cancer (OR per 1 ng/g increase: 0.78; 95% CI: 0.62–0.97), but no association was observed between other OC pesticides and thyroid cancer [55] (Table 6). Other studies investigating agricultural exposure to pesticides used the American Agricultural Health Study (AHS), which investigates health issues associated with agricultural exposure using questionnaires in a cohort of 89,655 pesticide applicators and their spouses in Iowa and North Carolina [106]. Lerro et al. (2020) used the AHS to investigate the risk of thyroid cancer among male pesticide users and found a positive association between the OC insecticide lindane and thyroid cancer (HR: 1.74; 95% CI: 1.06–2.84) [107]. Louis et al. used the AHS to look at different OC insecticides, but found no significant association between exposure and thyroid cancer among female spouses to insecticide applicators [108]. Saracci et al. investigated standardized mortality rates in a historical cohort study using an international register of 18,910 workers exposed to chlorophenoxy herbicides and chlorophenols from ten different countries. Results suggested an increased risk of thyroid cancer mortality associated with exposure (SMR: 357; 95% CI: 97–924), although the results were based on a small number of deaths (*n* = 4) [109]. Grimalt et al. investigated the mortality and cancer incidence in residents of Flix (Spain), a village located near an OC-compounds (HCB) factory where an unusually high level of HCB had previously been reported. Results showed a higher incidence of thyroid cancer in males but not in females: SIR: 6.7 (95% CI; 1.6–28) and SIR: 1.0 (95% CI: 0.14–7.4), respectively [110]. Ruder et al. investigated cancer mortality among workers exposed to PCP compared to the general US population but found no cases of associated thyroid mortality [111]. Deziel et al. (2020) found no significant association between OC pesticides and PTC among 250 female incident cases and 250 controls in Connecticut [56].

In summary, there is not enough evidence to conclude an association between exposure to OC pesticides and thyroid cancer. In accordance with findings on other endocrine cancers, future studies need to explore exposure to OCs at critical developmental periods in early life.

### 3.8. Organophosphate Pesticides

Following the ban on OC pesticides, countries switched to using non-persistent organophosphate (OP) pesticides. OP pesticides are used for agriculture, gardens and veterinary practices [112]. Most OP pesticides have a relatively short half-life ranging from hours to weeks [113]. Currently, 36 different types of OP pesticides are registered for use in the US, and all can cause acute and chronic toxicity [112]. Although they have a lower toxicity compared to the persistent OC pesticides, several OP pesticides are highly toxic such as carbophenothion, methyl parathion and ethyl parathion [112,114]. Therefore, various OP pesticides have been banned in the last decade due to toxicity and potentially adverse effects on the environment, animals and human health [112]. Diazinon and chloryporyfis were banned in 2001 for residential purposes but used with restrictions in agriculture [112,114]. Parathion was banned in 2003 for any use including agriculture. Malathion is currently used in the US for mosquito control and to kill insects on agricultural crops [115]. Due to the increasing health concerns, malathion has been controlled by the Food and Drug Administration (FDA) and the EPA [112,116]. Currently, eight parts per million (ppm) is allowed on crops used as foods [112]. In addition, 12 h must pass between applying and entry to avoid accidental exposure [112]. 

OP pesticides have several carcinogenic mechanisms as evaluated in multiple in-vitro studies. Chronic OP pesticide poisoning has cholinergic effects that downregulate aspects of the innate and adaptive immune system, creating a fertile environment for cancers to develop [117]. With regards to the innate immune system, OP pesticides downregulate serum esterase enzymes which in turn dampens complement and thrombin reactions. In addition, OP pesticide alteration of antigen-presenting cells, such as macrophages, may contribute to a blinding of the immune system to early antigens on tumor cells. With regards to the adaptive immune response, OP pesticides suppress cytotoxic T-cell activity [117]. In addition to the immunomodulatory effects, some OP pesticides including malathion, augment ROS in rats, a well-known contributing factor to many cancers due to mitochondrial and DNA damage [118]. The EPA has determined that there is suggestive evidence of carcinogenicity for malathion in animals but not in humans [33]. IARC has similarly classified malathion as probable carcinogenic to humans [31].

Only one known study has investigated the association between OP pesticides and thyroid cancer. Lerro et al. (2015) evaluated the use of OP pesticides and the risk of different cancer types among pesticide applicators using the AHS cohort, and found that malathion was associated with an increased risk of thyroid cancer in spouses of pesticide applicators (RR = 2.04, 95% CI 1.14–3.36) [119] (Table 6).

### 3.9. Other Pesticides

Other pesticides that have been assessed for their possible toxicity are alachlor and triazine (atrazine). Alachlor is an herbicide used for weed control on field corn, soybeans and peanuts [120]. Alachlor was introduced in 1969 but has been restricted due to its possible negative health effects on humans and wildlife [120]. The EPA has classified alachlor as a likely human carcinogen after evaluating carcinogenic activity in rodents [33]. In animal studies, high doses of alachlor were associated with elevated TSH levels and an increased incidence of thyroid neoplasia in male Long-Evans rats, indicating a mediated process for the development of thyroid follicular cell tumors in rodents [121].

A cohort study using the AHS found an elevated but non-significant risk of thyroid cancer associated with alachlor exposure compared to non-exposure (RR: 1.63; 95% CI: 0.42–6.37) [122] (Table 7). Acquavella et al. similarly examined the use of alachlor and the risk of thyroid cancer among workers exposed to the pesticide through occupational contact. There were no deaths reported from thyroid cancer and the overall cancer incidence was not significantly different in alachlor workers compared to a control group however this study was conducted by the epidemiology department of the Monsanto Company [123].

Atrazine is a chlorotriazine used as herbicide mainly on corn fields in the US. It was banned in 2004 by the European Union [124]. Atrazine is not listed as carcinogenic to humans, although there has been evidence of an association between atrazine exposure and thyroid cancer in rodents [125]. Beane Freeman et al. investigated atrazine and cancer incidence among pesticide applicators using the AHS, and found a potentially increased risk of thyroid cancer, although the results were based on a small number of cases (Intensity-weighted lifetime days of use in the highest quartile versus lowest quartile: RR: 4.30 (95% CI 1.19–15.57)) [126]. Lerro et al. (2016) used the AHS and found that spouses who reported ever using atrazine had no increased risk of developing thyroid cancer (RR: 2.00, 95% CI 0.88–4.57) [127].

Lerro et al. (2016) also investigated the association between thyroid cancer and other pesticides and found a significant association with exposure to the pesticide dicamba (RR: 2.34, 95% CI 1.03–5.35) and a non-significant association with metolachlor (RR: 2.22, 95% CI 0.92–5.35) [127].

In a more recent study using the AHS among male pesticide users, Lerro et al. (2018) found a positive association between the fungicide metalaxyl (HR: 2.03; 95% CI: 1.16–3.52) and thyroid cancer. In addition, there was an inverse association between the herbicide chlorimuron-ethyl (HR: 0.52; 95% CI: 0.28–0.96) and papillary thyroid cancer. Furthermore, high exposure to the insecticide carbaryl was inversely associated with thyroid cancer risk. (>median intensity-weighted days HR: 0.20; 95% CI: 0.08–0.53) [55].

N-methyl carbamate (carbamates) insecticides have been used in the US and worldwide for residential and agriculture use [128]. Carbamates also inhibit the acetylcholinesterase enzyme (AChE)—similar to OP pesticides—however, the effects are reversible and carbamates usually clear within 48 h. In addition, carbamates are more degraded than OP pesticides and have lower dermal toxicities [128,129]. Currently, the human health effects from carbamates are unknown. Some studies have found evidence of disrupted spermatogenesis and changes in epididymis related to carbamate exposure [130]. CDC has determined that the carbamate propoxur is a probable human carcinogen, based on studies showing bladder cancer in rats [33,70]. Furthermore, carbamates have been shown to alter gene expression and down-regulation of the antioxidant defense system, causing adverse effects on the dopamine system and inducing neurotoxicity and carcinogenesis [131]. In addition, mechanisms of carbamate insecticide-induced carcinogenesis may include damage and interference with the DNA repair process as well as oxidative stress. An animal study demonstrated that carbamates produced thyroid tumors in rodents [131].

Nordby et al. investigated the risk of thyroid cancer in farmers’ families exposed to mancozeb, a dithiocarbamate, using the central population register in Norway (*n* = 105,403 female farmers, *n* = 131,243 male farmers) [132]. Overall, 319 thyroid cancer cases were identified, but no significant association between exposure to mancozeb, using potato farming as measure of exposure, and the risk of thyroid cancer (RR: 0.87; 95% CI: 0.69–1.19) or papillary thyroid cancer (RR: 0.89; 95% CI: 0.67–1.18) was found. When using fungal forecasts as predictor of mancozeb use, no association was found between exposure and thyroid cancer risk (RR: 1.27 (95% CI: 0.83–1.93) when comparing 6–13 fungal forecast seasons to zero fungal forecast seasons [132].

## 4. Discussion

The incidence of thyroid cancer has steadily increased in recent decades, a period in which humans have been increasingly exposed to a large number of suspected EDCs with possibly carcinogenic effects [133].

EDCs are known to interfere with endocrine signaling at cellular and molecular levels, therefore potentially resulting in cancers of hormone sensitive organs, including breast, prostate, testis and thyroid. Certain synthetic estrogen-like EDCs (e.g., DDT, BPA) have been identified as potential risk factor for breast cancer, chlorinated pesticides and BPA are potentially associated with an increased risk of prostate cancer, and PFOA is suspected to be associated with in increased risk of testicular cancer [133]. The present review demonstrates that further research is urgently needed to evaluate the different EDCs and their potential carcinogenic effect on the thyroid gland as information is currently lacking for almost all known EDCs. Most EDCs have been studied sporadically and results are not consistent. Overall, the current literature suggests that exposure to certain congeners of flame retardants, PCBs, and phthalates as well as certain pesticides may potentially be associated with an increased risk of thyroid cancer.

It has been reported in previous studies that certain populations are more sensitive to EDCs exposure, including pregnant women and infants/toddlers, because organs are formed or certain endocrine feedback mechanism are not yet matured. EDCs exposure during pregnancy has been associated with many diseases in later life, such as obesity and metabolic diseases as well as certain reproductive cancers (e.g., DDT exposure and breast cancer, diethylstilbestrol (DES) exposure and vaginal cancer) [80], Moreover, transgenerational effects have been observed in generations not directly exposed to the EDCs due to epigenetic changes. This highlights that research investigating EDC exposure in these vulnerable populations is urgently needed.

A limitation of most studies to date is a cross-sectional study design meaning that the temporality of the association cannot be assessed, showing the need for longitudinal studies. Moreover, single exposure assessments disregard the possibility that exposures change over time. Furthermore, different studies used different measures to assess exposure, including urine, serum or dust samples or questionnaires, which potentially affected the accuracy of exposure assessment (e.g., recall bias when using questionnaires) and prevents combining the results of the different studies. Generalizability of the results of current studies to the general population is limited because most studies included specific study populations. Lastly, future studies assessing the association between EDC exposure and thyroid cancer need to control for potential confounders, e.g., occupational, lifestyle and dietary factors, and should explore the potential additive effect of exposure to multiple EDCs.

The current overview shows the urgent need to investigate the role of EDC exposure in the worldwide thyroid cancer burden. Longitudinal population-based exposure studies as well as studies of vulnerable populations are critical to increase the level of knowledge on the potential hazards of certain chemicals, shape regulatory actions and policies, and ultimately improve population health.

## Figures and Tables

**Table 1:** Association between exposure to flame retardants and thyroid cancer. Abbreviations: BB: brominated biphenyl; BCIPHIPP: 1-hydroxy-2-propyl bis(1-chloro-2-propyl) phosphate; BCIPP: bis(1,3-dichloro-2-propyl) phosphate; BDCIPP: bis(1,3-dichloro-2-propyl) phosphate; BDE: brominated diphenyl ethers; CI: confidence interval; DPHP: diphenyl phosphate; EDC: endocrine disrupting chemicals; IPDPP: isopropylated triphenyl phosphate; OR: Odd ratio; PBB: polybrominated biphenyl; PBDE: polybrominated diphenyl ethers; PFR: organophosphate flame retardants; PTC: papillary thyroid cancer; TBB: 2-ethylhexyl-2,3,4,5 tetrabromobenzoate; TBPH: bis(2-ethylhexyl)-2,3,4,5-tetrabromophthalate; TCEP: tris(2-chloroethyl) phosphate; TCIPP: tris(1-chloro-2-propyl) phosphate; TPHP: triphenyl phosphate.

Author (Year)	EDC Type (Congeners)	Country	Study Design	Investigated Population (*n*)	Measurement of Exposure	Association with Thyroid Cancer
Hoffman et al. (2017) [34]	Flame retardants (TBB, TBPH, TPHP, TCEP, TCIPP, BDE-47, BDE-99, BDE-100, BDE-153, BDE-154, BDE-209)	United States	Case-control	PTC cases from the Duke Cancer Institute (*n* = 70) Matched controls from the Duke Health system and surrounding communities (*n* = 70)	Dust samples.	**High (below median value) vs. Low exposure (above median value):** TBB: OR: 0.62 (95% CI: 0.29–1.31) TBPH: OR: 1.22 (95% CI: 0.56–2.65) TPHP: OR: 2.07 (95% CI: 0.94–4.56) **TCEP: OR: 2.42 (95% CI: 1.10–5.33)** TCIPP: OR:0.92 (95% CI: 0.43–1.97) BDE-47: OR: 0.80 (95% CI: 0.38–1.70) BDE-99: OR: 0.75 (95% CI: 0.36–1.59) BDE-100: OR: 0.88 (95% CI: 0.42–1.87) BDE-153: OR: 0.77 (95% CI: 0.37–1.63) BDE-154: OR: 0.80 (95% CI: 0.38–1.70) **BDE-209: OR: 2.29; 95% CI: 1.03–5.08)**
Huang et al. (2020) [35]	PBDEs (BDE-28, BDE-47, BDE-85, BDE-99, BDE-100, BDe-153, BDE-154) PBBs (BB-153)	United States	Nested case-control	United States Department of Defense cohort. (*n* = 1484) PTC cases (*n* = 742) Matched controls (*n* = 742)	Lipid-adjusted serum concentrations	**Third tertile vs. below limit of detection:** **Total population** **BDE-28: OR: 2.09 (95% CI: 1.02–4.15)** BDE-47: OR: 1.00 (95% CI: 0.41–2.44) BDE-85: OR: 1.76 (95% CI: 0.57–5.47) BDE-99: OR: 0.86 (95% CI: 0.39–1.87) BDE-100: OR: 0.67 (95% CI: 0.27–1.65) BDE- 153: OR: 0.94 (95% CI: 0.55–1.61) BDE-154: OR: 0.61 (95% CI: 0.19–1.99) BB-153: OR: 0.92 (95% CI: 0.65–1.31)
Aschebrook et al. (2015) [36]	PBDEs (BDE-47, BDE-99, BDE-100, BDE-153)	United States	Nested case-control	Prostate, Lung, Colorectal and Ovarian Cancer Screening Trial (*n* = 312) Thyroid cancer cases (*n* = 104) Matched controls (*n* = 208)	Lipid-adjusted serum samples	**Continuous:** BDE-47: OR: 0.95 (95% CI: 0.80–1.12) BDE-99: OR: 0.95 (95% CI: 0.81–1.11) BDE-100: OR: 0.96 (95% CI: 0.84–1.09) BDE-153: OR: 0.96 (95% CI: 0.82–1.11) Total PBDE: OR: 0.94 (95% CI: 0.79–1.11)
Deziel et al. (2019) [37]	PBDEs (BDE-28, BDE-47, BDE-85, BDE-99, BDE-100, BDE-153, BDE-154, BDE-183, BDE-209) PBBs (BB-153)	United States	Case-control	Women identified through Yale Cancer Center’s Rapid Case Ascertainment Shared Resources (*n* = 500) PTC cases (*n* = 250) Age- matched controls (*n* = 250)	Lipid-adjusted serum concentrations	**Continuous:** BDE-28: OR: 0.94 (95% CI: 0.78–1.13) BDE-47: OR: 0.89 (95% CI: 0.72–1.10) BDE-99: OR: 0.91 (95% CI: 0.74–1.12) BDE-100: OR: 1.05 (95% CI: 0.87–1.26) BDE-153: OR: 1.08 (95% CI: 0.90–1.30) BDE-209: OR: 0.87 (95% CI: 0.71–1.06) BB-153: OR: 1.15 (95% CI: 0.88–1.52) **Above LOD vs. below LOD:** BDE-85: OR: 0.71 (95% CI: 0.48–1.05) BDE-154: OR: 0.78 (95% CI: 0.53–1.13) BDE-183: OR: 0.74 (95% CI: 0.49–1.12)
Deziel et al. (2018) [39]	Organophosphate flame retardants (BCIPP. DPHP, BDCIPP, IPDPP, BCIPHIPP)	United States	Case-control	Women identified through Yale Cancer Center’s Rapid Case Ascertainment Shared Resources (*n* = 200) PTC cases (*n* = 100) Matched controls (*n* = 100)	Interviews and urine samples	**Continuous:** BCIPP: OR: 0.89 (95% CI: 0.76–1.04) DPHP: OR: 0.99 995% CI: 0.74–1.31) BDCIPP: OR: 1.07 (95% CI: 0.85–1.34) IPDPP: OR: 1.06 (95% CI: 0.75–1.48) BCIPHIPP: OR: 0.82 (95% CI: 0.65–1.01) Total PFR: OR: 0.93 (95% CI: 0.65–1.33)

**Table 2:** Association between exposure to PCBs and thyroid cancer. Abbreviations: CI: confidence interval; EDC: endocrine disrupting chemicals; OR: odds ratio; PCB: polychlorinated biphenyls; SMR: standardized mortality ratio.

Author (Year)	EDC Type (Congeners)	Country	Study Design	Investigated Population	Measurement of Exposure	Association with Thyroid Cancer
Zhuo et al. (2018) [54]	PCBs (PCB-28, PCB-74, PCB-99, PCB-105, PCB-118, PCB-138/158, PCB-146, PCB-153, PCB-156, PCB-157, PCB-167, PCB-170, PCB-178, PCB-180, PCB-183, PCB-187, PCB-194, PCB-196/203, PCB-199, PCB-206, PCB-209)	United States	Nested case-control	Department of Defense Automated Central Tumor Registry (ACTUR) and Defense Medical Surveillance System (DMSS) (*n* = 1484) PTC cases = 742 Matched controls = 742	Lipid-adjusted serum concentrations	**Fourth quartile vs. first quartile:** PCB-28: OR: 1.04 (95% CI: 0.62–1.76) PCB-74: OR: 1.24 (95% CI: 0.50–3.09) PCB-99: OR: 1.05 (95% CI: 0.68–1.62) PCB-105: OR: 1.02 (95% CI: 0.62–1.70) **PCB-118: OR: 1.55 (95% CI: 1.01–2.38)** PCB-138/158: OR: 1.07 (95% CI: 0.65–1.76) PCB-146: OR: 0.81 (95% CI: 0.48–1.38) PCB-153: OR: 1.01 (95% CI: 0.61–1.68) PCB-156: OR: 1.15 (95% CI: 0.67–1.95) PCB-157: OR: 1.44 (95% CI: 0.63–3.29) PCB-167: OR: 1.90 (95% CI: 0.74–4.85) PCB-170: OR: 0.92 (95% CI: 0.53–1.59) PCB-178: OR: 0.79 (95% CI: 0.34–1.88) PCB-180: OR: 0.80 (95% CI: 0.47–1.35) PCB-183: OR: 0.73 (95% CI: 0.39–1.35) PCB-187: OR: 0.89 (95% CI: 0.55–1.43) PCB-194: OR: 0.77 (95% CI: 0.43–1.36) PCB-196/203: OR: 1.08 (95% CI: 0.62–1.88) PCB-199: OR: 1.15 (95% CI: 0.68–1.95) PCB-206: OR: 1.57 (95% CI: 0.81–3.04) PCB-209: OR: 1.81 (95% CI: 0.83–3.97)
Lerro et al. (2018) [55]	PCBs (PCB-28, PCB-44, PCB-49, PCB- 52, PCB-66, PCB-74, PCB-87, PCB-99, PCB-101, PCB-105, PCB-110, PCB-114, PCB-118; PCB-128, PCB-138/158, PCB-146, PCB-149, PCB-151, PCB-153, PCB-156, PCB-157, PCB-167, PCB-170, PCB-172, PCB-177, PCB-178, PCB-180, PCB-183, PCB-187, PCB-189, PCB-194, PCB-195, PCB-196/203, PCB-199, PCB-206, PCB-209)	Norway	Nested case-control	Norwegian Janus Serum Bank cohort (*n* = 324) Thyroid cancer cases (*n* = 108) Controls (*n* = 216)	Blood samples	**Continuous:** PCB-28:OR: 1.05 (95% CI:0.70–1.58) PCB-44: OR: 0.97 (95% CI: 0.79–1.19) PCB-49: OR: 0.93 (95% CI: 0.67–1.30) PCB-52: OR: 0.95 (95% CI: 0.80–1.13) PCB-66: OR: 0.90 (95% CI: 0.65–1.24) PCB-74: OR: 0.85 (95% CI: 0.69–1.05) PCB-87: OR: 0.71 (95% CI: 0.33–1.55) PCB-99: OR: 0.85 (95% CI: 0.71–1.00) PCB-101: OR: 0.91 (95% CI: 0.67–1.23) PCB-105: OR: 0.83 (95% CI: 0.62–1.12) PCB-110: OR: 0.93 (95% CI: 0.65–1.33) PCB-114: OR: 0.78 (95% CI: 0.62–0.97) PCB-118: OR: 0.96 (95% CI: 0.89–1.04) PCB-128: OR: 0.96 (95% CI: 0.87–1.06) PCB-138/158: OR: 0.86 (5% CI: 0.64–1.15) PCB-146: OR: 0.94 (95% CI: 0.80–1.11) PCB-149: OR: 0.95 (95% CI: 0.87–1.03) PCB-151: OR: 0.98 (95% CI: 0.89–1.07) PCB-153: OR: 0.91 (95% CI: 0.72–1.14) PCB-156: OR: 0.90 (95% CI: 0.71–1.13) PCB-157: OR: 0.96 (95% CI: 0.87–1.06) PCB-167: OR: 0.98 (95% CI: 0.93–1.04) PCB-170: OR: 0.96 (95% CI: 0.87–1.05) PCB-172: OR: 0.98 (95% CI: 0.92–1.04) PCB-177: OR: 0.96 (95% CI: 0.92–1.01) PCB-178: OR: 0.98 (95% CI: 0.93–1.03) PCB-180: OR: 0.86 (95% CI: 0.58–1.27) PCB-183: OR: 0.85 (95% CI: 0.64–1.14) PCB-187: OR: 0.94 (95% CI: 0.84–1.06) PCB-189: OR: 0.63 (95% CI: 0.12–3.45) PCB-194: OR: 0.88 (95% CI: 0.67–1.15) PCB-195: OR: 0.91 (95% CI: 0.81–1.02) PCB-196/203: OR: 0.79 (95% CI: 0.59–1.04) PCB-199: OR: 0.77 (95% CI: 0.58–1.03) PCB-206: OR: 0.95 (95% CI: 0.88–1.02) PCB-209: OR: 1.00 (95% CI: 0.93–1.08) Total: OR: 0.96 (95% CI: 0.90–1.02)
Deziel et al. (2020) [56]	PCB-28, PCB-66, PCB-74, PCB-99, PCB-105, PCB-114, PCB-118, PCB-138 & 158, PCB-146, PCB-153, PCB-156, PCB-157, PCB-167, PCB-170, PCB-178, PCB-180, PCB-183, PCB-187, PCB-189, PCB-194, PCB-196 & 203, PCB-199, PCB-206, PCB-209,	United States	Case control	Incident female PTC cases (*n* = 250) Female controls (*n* = 250)	Interviews and serum samples	PCB-28: OR 0.89 (95% CI: 0.77–1.04) PCB-66: OR: 0.95 (95% CI: 0.84–1.06) PCB-74: OR: 0.94 (95% CI: 0.74–1.18) PCB-99: OR: 0.99 (95% CI: 0.88–1.10) PCB-105: OR: 0.98 (95% CI: 0.91–1.05) PCB-114: OR: 1.01 (95% CI: 0.82–1.25) PCB-118: OR: 0.99 (95% CI: 0.88–1.10) PCB-138 & 158: OR: 0.97 (95% CI 0.81–1.17) PCB-146: OR: 0.95 (95% CI: 0.76.-1.29) PCB-153: OR: 1.00 (95% CI: 0.78–1.29) PCB-156: OR: 0.99 (95% CI: 0.76–1.29) PCB-157: OR: 0.97 (95% CI: 0.75–1.25) PCB-167: OR: 0.99 (95% CI: 0.79–1.23) PCB-170: OR: 0.96 (95% CI: 0.70–1.33) PCB-178: OR: 1.04 (95% CI: 0.79–1.37) PCB-180: OR: 0.96 (95% CI: 0.70–1.33) PCB-183: OR: 1.04 (95% CI: 0.83–1.31) PCB-187: OR: 1.02 (95% CI: 0.81–1.27) PCB-189: OR: 0.90 (95% CI: 0.66–1.24) PCB-194: OR: 1.05 (95% CI: 0.75–1.45) PCB-196 & 203: OR: 1.12 (95% CI: 0.83–1.50) PCB-199: OR: 1.11 (95% CI: 0.84–1.45) PCB-206: OR: 1.07 (95% CI: 0.85–1.36) PCB-209: OR: 1.07 (95% CI: 0.89–1.30)
Ruder et al. (2014) [57]	PCBs	United States	Cohort	Capacitor-manufacturing workers exposed to PCBs at plants in Indiana, Massachusetts and New York (*n* = 24,865) Short term workers (*n* = 7647) Long term workers (*n* = 17,218)	Cumulative PCB exposure using plant-specific job-exposure matrices	**Standardized thyroid cancer mortality ratios:** Entire cohort: SMR: 0.52 (95% CI: 0.11–1.53) Short-term workers: SMR: 0.00 (95% CI: 0.00–2.76) Long-term workers: SMR: 0.68 (95% CI: 0.14–2.00)

**Table 3:** Association between exposure to phthalates and thyroid cancer. Abbreviations: CI: confidence interval; DEHP: di(2-ethylhexyl)phthalate; EDC: endocrine disrupting chemicals; MBP: monobutyl phthalate; MBzP: monobenzyl phthalate; MECPP: mono(2-ethyl-5-carboxypentyl) phthalate; MEHHP: mono (2-ethyl-5-hydroxyhexyl) phthalate; MEHP: mono-ethylhexyl phthalate; MEOHP: mono (2-ethyl-5-oxohexyl) phthalate; MEP: Monoethyl phthalate; MMP: mono-methyl phthalate; OR: odds ratio; PTC: papillary thyroid cancer; TC: thyroid cancer.

Author (Year)	EDC Type (Metabolites)	Study Design	Country	Investigated Population	Measurement	Association with Thyroid Cancer
Marotta et al. (2019) [70]	DEHP (MEHP)	Cross-sectional	Italy	Patients with differentiated TC (*n* = 28) Patients with benign thyroid nodules (*n* = 27)	Serum	MHEP: OR: 3.19 (95% CI: 0.85–11.87) (unadjusted) **DHEP: OR: 15.07 (95% CI: 1.59–142.13)**
Miao et al. (2020) [66]	DEHP (MBP, MEP, MEHP, MEOHP, MECPP, MEHHP)	Case-control	China	Cancer Hospital of Chinese Academy of Medical Sciences PTC cases (*n* = 111) Controls with non-PTC (*n* = 111)	Urinary phthalates metabolite concentrations	MBP: OR: 1.48 (95% CI: 0.98–2.24) MEP: OR: 1.40 (95% CI: 0.90–2.19) **MEHP: OR: 7.30 (95% CI: 2.17–24.56)** **MEOHP: OR: 2.07 (95% CI: 1.21–3.53)** **MECPP: OR: 3.11 (95% CI: 1.56–6.19)** **MEHHP: OR: 3.63 (95% CI: 1.69–7.74)** **Total DEHP: OR: 3.15 (95% CI: 1.64–7.49)**
Liu et al. (2020) [71]	Phthalate metabolites (MMP, MEP, MBP, MBzP, MEHHP, MEOHP, MEHP)	Case-control	China	Central Hospital of Wuhan, China Thyroid cancer cases (*n* = 144) Healthy adults (*n* = 144)	Creatinine–adjusted urinary phthalate metabolite concentrations	**Continuous:** **MMP: OR: 1.11 (95% CI: 1.01–1.22)** MEP: OR: 0.97 (95% CI: 0.84–1.13) **MBP: OR: 0.45 (95% CI: 0.34–0.60)** **MBzP: OR: 0.71 (95% CI: 0.60–0.85)** MEOHP: OR: 1.09 (95% CI: 0.84–1.42) MEHHP: OR: 1.53; 95% CI: 1.19–1.96) MEHP: OR: 1.46; 95% CI: 1.09–1.91)

**Table 4:** Association between exposure to BPA and thyroid cancer. Abbreviations: BPA: bisphenol A; BADGE: Bisphenol A diglycidyl ether; BPAF: Bisphenol AF; BPB: Bisphenol B; BPE: Bisphenol E; BPF: Bisphenol F; BPS: Bisphenol S; CI: confidence interval; EDC: endocrine disrupting chemicals; PTC: papillary thyroid cancer; TC: thyroid cancer.

Author (Year)	EDC Type	Study Design	Country	Investigated Population	Measurement of Exposure	Association with Thyroid Cancer
Zhou et al. (2017) [81]	BPA	Cross-sectional	China	Qilu Hospital of Shandong University PTC (*n* = 53) Healthy volunteers (*n* = 65)	Urinary BPA concentrations	**Higher urinary BPA (>2.84 ng/mL) vs. lower urinary BPA:** **OR: 3.57 (95% CI: 1.37–9.30)**
Marotta et al. (2019) [70]	BPA, BPS, BPF, BPE, BPB, BPAF, BADGE	Cross-sectional	Italy	Patients with differentiated TC (*n* = 28) Patients with benign thyroid nodules (*n* = 27)	Serum	**Unadjusted analysis:** BPA: OR: 3.17 (95% CI: 0.67–20.34) BPS: OR: 10.1 (95% CI: 0.51–197.32) BPF: OR: 5.18 (95% CI: 0.23–111.22) BPB: OR: 1.50 (95% CI: 0.46–4.83) BAGDE: OR: 2.66 (5% CI: 0.61–11.64) **Adjusted analysis:** BPE: OR: 3.62 (95% CI: 0.69–18.95) **BPAF: OR: 15.07 (95% CI: 1.59–142.13)**

**Table 5:** Association between exposure to PFAS and thyroid cancer. Abbreviations: CI: confidence interval; EDC: endocrine disrupting chemicals; HR: hazard ratio; NDI: national death index; OR: odds ratio; PFOA: perfluorooctanoic acid; RR: risk ratio; SMR: standardized mortality ratio.

Author (Year)	EDC Type	Study Design	Country	Investigated Population	Measurement of Exposure	Association with Thyroid Cancer
Barry et al. (2013) [89]	PFOA	Cohort	United States	C8 Health Project (*n* = 32,254)	Estimated PFOA serum levels	No lag: HR: 1.10 (95% CI 0.95–1.20) 10-year lag: HR: 1.04 (95% CI: 0.89–1.20)
Vieria et al. (2013) [90]	PFOA	Case-control	United States	Incident cancer cases among residents living near the DuPont Teflon-manufacturing plant in Parkersburg, West Virginia (*n* = 19,716) using cancer registry data	Estimated individual-level annual PFOA serum exposure categories	Very high: OR: 0.8 (95% CI: 0.2–3.5) High: OR: 0.7 (95% CI: 0.2–2.1) Medium: OR: 0.9 (95% CI: 0.4–2.3) Low: OR: 1.2 (95% CI: 0.8–1.7)
Olsen et al. (2004) [91]	PFOA	Case-control	United States	Fluorochemical production facility employees (*n* = 652 cases) and film plant employees (*n* = 659 controls)	Health claims data	RR (95% CI): unable to calculate
Leonard et al. (2008) [92]	PFOA	Case-control	United States	Workers at polymer manufacturing facility (*n* = 6027) Workers at other DuPont facilities (*n* = 72,882)	Causes of death (Dupont Epidemiology Registry) or (NDI Plus)	SMR: 628.6 (95% CI: 129.7; 1836.9)

**Table 6:** Association between exposure to organochlorine and organophosphate pesticides and thyroid cancer. Abbreviations: AHS: Agricultural Health Study; CI: confidence interval; DDE: Dichlorodiphenyldichloroethylene; DDT: dichlorodiphenyltrichloroethane; EDC: endocrine disrupting chemicals; HCB: Hexachlorobenzene; HCCH: hexachlorocyclohexanes; HR: hazard ratio; OC: organochlorine; OP: organophosphate; OR: odds ratio; PCB: polychlorinated biphenyl; PCP: Pentachlorophenol; PTC: papillary thyroid cancer; RR: risk ratio; SIR: standardized incidence ratio; SMR: standardized mortality ratio.

Author (Year)	EDC Type	Study Design	Country	Investigated Population	Measurement of Exposure	Association with Thyroid Cancer
Lerro et al. (2018) [55]	Organochlorine Pesticides (DDT metabolites)	Nested case-control	Norway	Norwegian Janus Serum Bank cohort Thyroid cancer cases (*n* = 108) Control (*n* = 216)	Blood samples	**Continuous:** *p,p’*-DDE: OR: 0.79 (95% CI: 0.64–0.97) *p,p’*-DDT: OR: 0.86 (95% CI: 0.71–1.05) *o,p’*-DDT: OR: 0.81 (95% CI: 0.64–1.02) Total DDT metabolites: OR: 0.80 (95% CI: 0.66–0.98)
Lerro et al. (2020) [107]	Lindane	Cohort	United States	AHS cohort (*n* = 53,096)	Self-administered questionnaires on pesticide exposure	**HR: 1.74 (95% CI: 1.06–2.84)**
Louis et al. (2017) [108]	Organochlorine pesticides (Aldrin, chlordane, dieldrin, DDT, heptachlor, lindane, toxaphene)	Cohort	United States	Female spouses of pesticide applicators in the AHS (*n* = 32,345) No pesticide use (*n* = 26,718) Pesticide use (*n* = 2191)	Self-administered questionnaires on OC pesticide exposure	Ever versus never exposed: Any OC: RR: 0.66 (95% CI: 0.26–1.63) Chlordane: RR: 0.97 (95% CI: 0.36–2.67) Not more than 3 exposed thyroid cancer cases for other OC pesticides so RR not calculated.
Saracci et al. (1991) [109]	Chlorophenoxy herbicides and/or chlorophenols	Cohort	France	International register of production workers or sprayers form ten countries (*n* = 18,910) Exposed workers (*n* = 13,482) Probably exposed workers (*n* = 416) Non-exposed workers (*n* = 3951)	Questionnaires on exposure	SMR: 357 (95% CI: 97–914) (4 thyroid cancer-related deaths)
Grimalt et al. (1994) [110]	Hexachlorobenzene (HCB)	Cohort	Spain	Community exposed to HCB (*n* = 5003) Reference community	Average 24-h air levels of OC compounds (including HCB, PCB, pp’DDE, chloroform, carbon tetrachloride, trichloroethylene, tetrachloroethylene)	Male: SIR: 6.7 (95% CI: 1.6–28) Female: SIR: 1.0 (95% CI: 0.14–7.4)
Deziel et al. (2020) [56]	Organochlorine insecticides	Case control	United States	Incident female PTC cases (*n* = 250) Female controls (*n* = 250)	Interviews and serum samples	Hexachlorobenzene: OR: 1.02 (95% CI: 0.91–1.14) β-HCCH: OR: 1.00 (95% CI: 0.97–1.03) Oxychlordane: OR: 1.00 (95% CI: 0.74–1.36) *trans*-Nonachlor: OR: 0.94 (95% CI: 0.75–1.19) p,p’-DDE: OR: 0.97 (95% CI: 0.90–1.05) p,p’-DDT: OR: 0.99 (95% CI: 0.90–1.09) Mirex: OR: 1.02 (95% CI: 0.95–1.09)
Ruder et al. (2011) [111]	Pentachlorophenol (PCP)	Cohort	United States	PCP production workers from four plants in the National Institute for Occupational Safety and Health Dioxin Registry (*n* = 2122)	PCP exposure level from registry	No cases of thyroid cancer mortality reported
Lerro et al. (2015) [119]	Malathion, diazinon, chlorpyrifos, terbufos	Cohort	United States	Female spouses of pesticide applicators in the AHS Total (*n* = 29,325) OP pesticide use (*n* = 7589) No OP pesticide use (*n* = 21,736)	Self-administered questionnaires on OP pesticide exposure	**Ever versus never OP pesticide use:** **Malathion: RR: 2.04 (95% CI: 1.14–3.36)** Any OP pesticide: RR: 1.27 (95% CI: 0.70–2.30) Not more than 10 exposed thyroid cancer cases for other OP pesticides so RR not calculated.

**Table 7:** Association between exposure to other pesticides and thyroid cancer. Abbreviations: AHS: Agricultural Health Study; CI: confidence interval; EDC: endocrine disrupting chemicals; HR: hazard ratio; RR: risk ratio; SIR: standardized incidence ratio.

Author (Year)	EDC Type	Study Design	Country	Investigated Population	Measurement of Exposure	Association with Thyroid Cancer
Lee et al. (2004) [122]	Alachlor	Cohort	United States	AHS (*n* = 49,980) Alachlor exposed applicators (*n* = 26,510) Non-exposed applicators (*n* = 23,470)	Self-administered exposure assessment questionnaire	Alachlor exposed population: SIR: 1.26 (95% CI: 0.61–2.33) Alachlor unexposed population: SIR: 0.90 (95% CI: 0.33–1.97) Exposed vs. unexposed: RR: 1.63 (95% CI: 0.42–6.37)
Acquavella et al. (2004) [123]	Alachlor	Cohort	United States	Muscatine manufactoring workers Mortality analysis (*n* = 1206)	The State Health Registry of Iowa, Personnel records	No thyroid cancer case reported in this study
Beane Freeman et al. (2011) [126]	Atrazine	Cohort	United States	AHS (*n* = 57,310) Atrazine use (*n* = 17,305)	Self-administered questionnaires on pesticide exposure	Intensity-weighted lifetime days of use in the highest quartile versus lowest quartile: RR: 4.30 (95% CI: 1.19–15.57)
Lerro et al. (2016) [127]	Atrazine, dicamba, metolachlor carbaryl	Cohort	United States	AHS female spouses (*n* = 31,055) Thyroid cancer cases (*n* = 104)	Self-administered questionnaires on pesticide exposure	Atrazine: RR: 2.00 (95% CI: 0.88–4.57) Dicamba: RR: 2.34 (95% CI: 1.03–5.35) Metolachlor: RR: 2.22, 95% CI: 0.92–5.35) Carbaryl: RR: 0.61, 95% CI: 0.36–1.03
Lerro et al. (2018) [55]	Chlorimuron-ethyl, carbaryl, metalaxyl	Cohort	United States	AHS (*n* = 53,096)	Self-administered questionnaires on pesticide exposure	HR: 0.52 (95% CI: 0.28–0.96) (Papillary thyroid cancer) High exposure to Carboryl (>median intensity-weighted days HR: 0.30 (95% CI: 0.08–0.53)) Metalaxyl: HR: 2.03 (95% CI: 1.16–3.52)
Nordby et al. (2005) [132]	Mancozeb	Cohort	Norway	Central population register in Norway (*n* = 105,403 female farmers) (*n* = 131,243 male farmers) Thyroid cancer case: 319	Population registry	Potato farming (yes vs. no): Thyroid cancer: RR: 0.87 (95% CI 0.69–1.10) Papillary subgroup: RR: 0.89 (95% CI: 0.67–1.18) Fungal forecasts: 0 seasons: ref 1–2 seasons: RR: 1.01 (95% CI: 0.71–1.43) 3–5 seasons: RR: 1.16 (95% CI: 0.76–1.77) 6–13 seasons: RR: 1.27 (95% CI: 0.83–1.93)

## Data Availability

Not applicable.

## References

[1] Davies L., Welch H.G. (2006). Increasing incidence of thyroid cancer in the United States, 1973–2002. JAMA.

[2] Kitahara C.M., Sosa J.A. (2016). The changing incidence of thyroid cancer. Nat. Rev. Endocrinol..

[3] Fiore M., Oliveri Conti G., Caltabiano R., Buffone A., Zuccarello P., Cormaci L., Angel Cannizzaro M., Ferrante M. (2019). Role of emerging environmental risk factors in thyroid cancer: A brief review. Int. J. Environ. Res. Public Health.

[4] Wingren G.B., Axelson O. (1997). Occupational and environmental determinants for benign thyroid disease and follicular thyroid cancer. Int. J. Occup. Med. Environ. Health.

[5] Zoeller T.R., Dowling A.L., Herzig C.T., Iannacone E.A., Gauger K.J., Bansal R. (2002). Thyroid hormone, brain development, and the environment. Environ. Health Perspect..

[6] Camargo R.Y.A., Tomimori E.K., Neves S.C., Rubio I.G.S., Galrao A.L., Knobel M., Medeiros-Neto G. (2008). Thyroid and the environment: Exposure to excessive nutritional iodine increases the prevalence of thyroid disorders in Sao Paulo, Brazil. Eur. J. Endocrinol..

[7] Soto A.M., Sonnenschein C. (2010). Environmental causes of cancer: Endocrine disruptors as carcinogens. Nat. Rev. Endocrinol..

[8] Damstra T., Barlow S., Bergman A., Kavlock R., Van Der Kraak G. (2002). Global Assessment of the State-of-the-Science of Endocrine Disruptors.

[9] National Institute of Environmental Health Sciences Endocrine Disruptors and Your Health. https://www.niehs.nih.gov/health/materials/endocrine_disruptors_508.pdf.

[10] Dyer C.A. (2007). Heavy metals as endocrine-disrupting chemicals. Endocrine-Disrupting Chemicals.

[11] National Institute of Environmental Health Sciences Flame Retardants. https://www.niehs.nih.gov/health/topics/agents/flame_retardants/index.cfm.

[12] Chen S., Ma Y., Wang J., Chen D., Luo X., Mai B. (2009). Brominated flame retardants in children’s toys: Concentration, composition, and children’s exposure and risk assessment. Environ. Sci. Technol..

[13] Weil E.D., Levchik S.V. (2008). Flame retardants in commercial use or development for textiles. J. Fire Sci..

[14] Schreder E.D., Uding N., La Guardia M.J. (2016). Inhalation a significant exposure route for chlorinated organophosphate flame retardants. Chemosphere.

[15] Davis A., Harris D., Black M. (2019). A study of chemical exposure from consumer products used in residential environments. ASHRAE Trans..

[16] United States Environmental Protection Agency Emerging Contaminants- Polybrominated Diphenyls Ethers (PBDE) and Polybrominated Biphenyls (PBB). https://nepis.epa.gov/Exe/tiff2png.cgi/P1000L3S.PNG?-r+75+-g+7+D%3A%5CZYFILES%5CINDEX%20DATA%5C06THRU10%5CTIFF%5C00000138%5CP1000L3S.TIF.

[17] Centers for Disease Control and Prevention Polybrominated Biphenys (PBBs). https://www.atsdr.cdc.gov/toxfaqs/tfacts68.pdf.

[18] Centers for Disease Control and Prevention Polybrominated Diphenyl Ethers (PBDEs) and Polybrominated Biphenyls (PBBs). https://www.cdc.gov/biomonitoring/PBDEs_FactSheet.html.

[19] Dodson R.E., Perovich L.J., Covaci A., Van den Eede N., Ionas A.C., Dirtu A.C., Brody J.G., Rudel R.A. (2012). After the PBDE phase-out: A broad suite of flame retardants in repeat house dust samples from California. Environ. Sci. Technol..

[20] Oulhote Y., Chevrier J., Bouchard M.F. (2016). Exposure to polybrominated diphenyl ethers (PBDEs) and hypothyroidism in canadian women. Int. J. Clin. Endocrinol. Metab..

[21] Rosen D.H., Flanders W.D., Friede A., Humphrey H.E., Sinks T.H. (1995). Half-life of polybrominated biphenyl in human sera. Environ. Health Perspect..

[22] Ongono J.S., Dow C., Gambaretti J., Severi G., Boutron-Ruault M., Bonnet F., Mancini F.R. (2019). Dietary exposure to brominated flame retardants and risk of type 2 diabetes in the french E3N cohort. Environ. Int..

[23] Herbstman J.B., Sjödin A., Kurzon M., Lederman S.A., Jones R.S., Rauh V., Needham L.L., Tang D., Niedzwiecki M., Wang R.Y. (2010). Prenatal exposure to PBDEs and neurodevelopment. Environ. Health Perspect..

[24] Harley K.G., Marks A.R., Chevrier J., Bradman A., Sjödin A., Eskenazi B. (2010). PBDE concentrations in women’s serum and fecundability. Environ. Health Perspect..

[25] Zoeller R.T., Bergman A., Becher G., Bjerregaard P., Bornman R., Brandt I., Iguchi T., Jobling S., Kidd K.A., Kortenkamp A. (2014). A path forward in the debate over health impacts of endocrine disrupting chemicals. Environ. Health.

[26] Liu S., Zhao G., Li J., Zhao H., Wang Y., Chen J., Zhao H. (2017). Association of polybrominated diphenylethers (PBDEs) and hydroxylated metabolites (OH-PBDEs) serum levels with thyroid function in thyroid cancer patients. Environ. Res..

[27] Allen J.G., Gale S., Zoeller R.T., Spengler J.D., Birnbaum L., McNeely E. (2016). PBDE flame retardants, thyroid disease, and menopausal status in US women. Environ. Health.

[28] Mackenzie P.I., Owens I.S., Burchell B., Bock K.W., Bairoch A., Belanger A., Fournel-Gigleux S., Green M., Hum D.W., Iyanagi T. (1997). The UDP glycosyltransferase gene superfamily: Recommended nomenclature update based on evolutionary divergence. Pharm. Genom..

[29] Zhao X., Wang H., Li J., Shan Z., Teng W., Teng X. (2015). The correlation between polybrominated diphenyl ethers (PBDEs) and thyroid hormones in the general population: A meta-analysis. PLoS ONE.

[30] Lin H., Chin Y., Yang Y.S., Lai H., Wang-Peng J., Liu L.F., Tang H., Davis P.J. (2011). Thyroid hormone, cancer, and apoptosis. Compr. Physiol..

[31] International Agency for Research on Cancer Classifications. https://monographs.iarc.fr/agents-classified-by-the-iarc/.

[32] U.S. Department of Health and Human Services, National Toxicology Program 14th Report on Carcinogens. https://ntp.niehs.nih.gov/whatwestudy/assessments/cancer/roc/index.html.

[33] United States Environmental Protection Agency Risk Assessment for Carcinogenic Effects. https://www.epa.gov/fera/risk-assessment-carcinogenic-effects.

[34] Hoffman K., Lorenzo A., Butt C.M., Henderson B.B., Roman S.A., Scheri R.P., Stapleton H.M., Sosa J.A. (2017). Exposure to flame retardant chemicals and occurrence and severity of papillary thyroid cancer: A case-control study. Environ. Int..

[35] Huang H., Sjodin A., Chen Y., Ni X., Ma S., Yu H., Ward M.H., Udelsman R., Rusiecki J. (2020). Polybrominated diphenyl ethers, polybrominated biphenyls, and risk of papillary thyroid cancer: A nested case-control study. Am. J. Epidemiol..

[36] Aschebrook-Kilfoy B., DellaValle C.T., Purdue M., Kim C., Zhang Y., Sjodin A., Ward M.H. (2015). Polybrominated diphenyl ethers and thyroid cancer risk in the prostate, colorectal, lung, and ovarian cancer screening trial cohort. Am. J. Epidemiol..

[37] Deziel N.C., Alfonso-Garrido J., Warren J.L., Huang H., Sjodin A., Zhang Y. (2019). Exposure to polybrominated diphenyl ethers and a polybrominated biphenyl and risk of thyroid cancer in women: Single and multi-pollutant approaches. Cancer Epidemiol. Biomark. Prev..

[38] Hoffman K., Sosa J.A., Stapleton H.M. (2017). Do flame retardant chemicals increase the risk for thyroid dysregulation and cancer?. Curr. Opin. Oncol..

[39] Deziel N.C., Yi H., Stapleton H.M., Huang H., Zhao N., Zhang Y. (2018). A case-control study of exposure to organophosphate flame retardants and risk of thyroid cancer in women. BMC Cancer.

[40] Jacobson M.H., Darrow L.A., Barr D.B., Howards P.P., Lyles R.H., Terrell M.L., Smith A.K., Conneely K.N., Marder M.E. (2017). Serum polybrominated biphenyls (PBBs) and polychlorinated biphenyls (PCBs) and thyroid function among michigan adults several decades after the 1973–1974 PBB contamination of livestock feed. Environ. Health Perspect..

[41] Meador J.P. (1996). Environmental Contaminants in Wildlife: Interpreting Tissue Concentrations.

[42] Gore A.C., Chappell V.A., Fenton S.E., Flaws J.A., Nadal A., Prins G.S., Toppari J., Zoeller R.T. (2015). EDC-2: The Endocrine Society’s Second Scientific Statement on Endocrine-Disrupting Chemicals. Endocr. Rev..

[43] United States Environmental Protection Agency Polychlorinated Biphenyls. https://www.epa.gov/pcbs.

[44] Ritter R., Scheringer M., MacLeod M., Moeckel C., Jones K.C., Hungerbühler K. (2011). Intrinsic human elimination half-lives of polychlorinated biphenyls derived from the temporal evolution of cross-sectional biomonitoring data from the united kingdom. Environ. Health Perspect..

[45] Leng L., Li J., Luo X., Kim J., Li Y., Guo X., Chen X., Yang Q., Li G., Tang N. (2016). Polychlorinated biphenyls and breast cancer: A congener-specific meta-analysis. Environ. Int..

[46] Murray I.A., Patterson A.D., Perdew G.H. (2014). Aryl hydrocarbon receptor ligands in cancer: Friend and foe. Nat. Rev. Cancer.

[47] Lauby-Secretan B., Loomis D., Baan R., Ghissassi F.E., Bouvard V., Benbrahim-Tallaa L., Guha N., Grosse Y., Straif K. (2016). Use of mechanistic data in the IARC evaluations of the carcinogenicity of polychlorinated biphenyls and related compounds. Environ. Sci. Pollut. Res..

[48] Byrne J.J., Carbone J.P., Hanson E.A. (1987). Hypothyroidism and abnormalities in the kinetics of thyroid hormone metabolism in rats treated chronically with polychlorinated biphenyl and polybrominated biphenyl. Endocrinology.

[49] Benson K., Yang E., Dutton N., Sjodin A., Rosenbaum P.F., Pavuk M. (2018). Polychlorinated biphenyls, indicators of thyroid function and thyroid autoantibodies in the anniston community health survey I (ACHS-I). Chemosphere.

[50] Matsuura N., Uchiyama T., Tada H., Nakamura Y., Kondo N., Morito M., Fukushi M. (2001). Effects of dioxins and polychlorinated biphenyls (PCBs) on thyroid function in infants born in japan–the second report from research on environmental health. Chemosphere.

[51] Chevrier J., Eskenazi B., Holland N., Bradman A., Barr D.B. (2008). Effects of exposure to polychlorinated biphenyls and organochlorine pesticides on thyroid function during pregnancy. Am. J. Epidemiol..

[52] Hagmar L. (2003). Polychlorinated biphenyls and thyroid status in humans: A review. Thyroid.

[53] Montaño M., Cocco E., Guignard C., Marsh G., Hoffmann L., Bergman A., Gutleb A.C., Murk A.J. (2012). New approaches to assess the transthyretin binding capacity of bioactivated thyroid hormone disruptors. Toxicol. Sci..

[54] Zhuo H. (2018). Exposure to Polychlorinated Biphenyl (PCBs) and Risk of Papillary Thyroid Cancer (PTC): A Nested Case-Control Study. Ph.D. Thesis.

[55] Lerro C.C., Jones R.R., Langseth H., Grimsrud T.K., Engel L.S., Sjodin A., Choo-Wosoba H., Albert P., Ward M.H. (2018). A nested case-control study of polychlorinated biphenyls, organochlorine pesticides, and thyroid cancer in the janus serum bank cohort. Environ. Res..

[56] Deziel N.C., Warren J.L., Huang H., Zhou H., Sjodin A., Zhang Y. (2020). Exposure to polychlorinated biphenyls and organochlorine pesticides and thyroid cancer in connecticut women. Environ. Res..

[57] Ruder A.M., Hein M.J., Hopf N.B., Waters M.A. (2014). Mortality among 24,865 workers exposed to polychlorinated biphenyls (PCBs) in three electrical capacitor manufacturing plants: A ten-year update. Int. J. Hyg. Environ. Health.

[58] United States Environmental Protection Agency Phthalates. https://www.epa.gov/assessing-and-managing-chemicals-under-tsca/phthalates.

[59] Bornehag C., Sundell J., Weschler C.J., Sigsgaard T., Lundgren B., Hasselgren M., Hagerhed-Engman L. (2004). The association between asthma and allergic symptoms in children and phthalates in house dust: A nested case-control study. Environ. Health Perspect..

[60] Bertelsen R.J., Carlsen K.C.L., Calafat A.M., Hoppin J.A., Haland G., Mowinckel P., Carlsen K., Lovik M. (2013). Urinary biomarkers for phthalates associated with asthma in norwegian children. Environ. Health Perspect..

[61] Zuccarello P., Conti G.O., Cavallaro F., Copat C., Cristaldi A., Fiore M., Ferrante M. (2018). Implication of dietary phthalates in breast cancer. A systematic review. Food Chem. Toxicol..

[62] Swan S.H. (2008). Environmental phthalate exposure in relation to reproductive outcomes and other health endpoints in humans. Environ. Res..

[63] Kim M.J., Moon S., Oh B., Jung D., Choi K., Park Y.J. (2019). Association between diethylhexyl phthalate exposure and thyroid function: A meta-analysis. Thyroid.

[64] Ye H., Ha M., Yang M., Yue P., Xie Z., Liu C. (2017). Di2-ethylhexyl phthalate disrupts thyroid hormone homeostasis through activating the ras/akt/TRHr pathway and inducing hepatic enzymes. Sci. Rep..

[65] Huang H., Pan W., Chang J., Chiang H., Guo Y.L. (2017). Does exposure to phthalates influence thyroid function and growth hormone homeostasis? the taiwan environmental survey for toxicants (TEST) 2013. Environ. Res..

[66] Miao H., Liu X., Li J., Zhang L., Zhao Y., Liu S., Ni S., Wu Y. (2020). Associations of urinary phthalate metabolites with risk of papillary thyroid cancer. Chemosphere.

[67] Wu Y., Li J., Yan B., Zhu Y., Liu X., Chen M., Li D., Lee C., Yang X., Ma P. (2017). Oral exposure to dibutyl phthalate exacerbates chronic lymphocytic thyroiditis through oxidative stress in female wistar rats. Sci. Rep..

[68] Erkekoglu P., Kocer-Gumusel B., Kizilgun M., Hininger-Favier I., Rachidi W., Roussel A., Favier A., Hincal F. (2012). Thyroidal effects of di-(2-ethylhexyl) phthalate in rats of different selenium status. J. Environ. Pathol. Toxicol. Oncol..

[69] Centers for Disease Control and Prevention Health Effects of Exposures to Substances and Carcinogens. https://www.atsdr.cdc.gov/substances/ToxOrganSystems.asp.

[70] Marotta V., Russo G., Gambardella C., Grasso M., La Sala D., Chiofalo M.G., D’Anna R., Puzziello A., Docimo G., Masone S. (2019). Human exposure to bisphenol AF and diethylhexylphthalate increases susceptibility to develop differentiated thyroid cancer in patients with thyroid nodules. Chemosphere.

[71] Liu C., Deng Y., Zheng T., Yang P., Jiang X., Liu E., Miao X., Wang L., Jiang M., Zeng Q. (2020). Urinary biomarkers of phthalates exposure and risks of thyroid cancer and benign nodule. J. Hazard. Mater..

[72] National Institute of Environmental Health Sciences Bisphenol A (BPA). https://www.niehs.nih.gov/health/topics/agents/sya-bpa/index.cfm.

[73] Vandenberg L.N., Hauser R., Marcus M., Olea N., Welshons W.V. (2007). Human exposure to bisphenol A (BPA). Reprod. Toxicol..

[74] Huo X., Chen D., He Y., Zhu W., Zhou W., Zhang J. (2015). Bisphenol-A and female infertility: A possible role of gene-environment interactions. Int. J. Environ. Res. Public Health.

[75] Ziv-Gal A., Flaws J.A. (2016). Evidence for bisphenol A-induced female infertility: A review (2007–2016). Fertil. Steril..

[76] Kim M.J., Park Y.J. (2019). Bisphenols and thyroid hormone. Endocrinol. Metab..

[77] Cuomo D., Porreca I., Cobellis G., Tarallo R., Nassa G., Falco G., Nardone A., Rizzo R., Mallardo M., Ambrosino C. (2017). Carcinogenic risk and bisphenol A exposure: A focus on molecular aspects in endoderm derived glands. Mol. Cell Endocrinol..

[78] Gentilcore D., Porreca I., Rizzo F., Ganbaatar E., Carchia E., Mallardo M., De Felice M., Ambrosino C. (2013). Bisphenol A interferes with thyroid specific gene expression. Toxicology.

[79] Porreca I., Severino L.U., D’Angelo F., Cuomo D., Ceccarelli M., Altucci L., Amendola E., Nebbioso A., Mallardo M., De Felice M. (2016). “Stockpile” of slight transcriptomic changes determines the indirect genotoxicity of low-dose BPA in thyroid cells. PLoS ONE.

[80] Demeneix B., Slama R. (2019). Endocrine disruptors: From scientific evidence to human health protection. Eur. Parliam. Rep..

[81] Zhou Z., Zhang J., Jiang F., Xie Y., Zhang X., Jiang L. (2017). Higher urinary bisphenol A concentration and excessive iodine intake are associated with nodular goiter and papillary thyroid carcinoma. Biosci. Rep..

[82] National Institute of Environmental Health Sciences Per- and Polyfluoroalkyl Substances (PFAS). https://www.epa.gov/pfas.

[83] Fromme H., Tittlemier S.A., Völkel W., Wilhelm M., Twardella D. (2009). Perfluorinated compounds–exposure assessment for the general population in western countries. Int. J. Hyg. Environ. Health.

[84] Bergman Å., Heindel J.J., Jobling S., Kidd K., Zoeller T.R. (2013). State of the Science of Endocrine Disrupting Chemicals 2012.

[85] Li Y., Fletcher T., Mucs D., Scottm K., Lindh C.H., Tallving P., Jakobsson K. (2018). Half-lives of PFOS, PFHxS and PFOA after end of exposure to contaminated drinking water. Occup. Environ. Med..

[86] Coperchini F., Awwad O., Rotondi M., Santini F., Imbriani M., Chiovato L. (2017). Thyroid disruption by perfluorooctane sulfonate (PFOS) and perfluorooctanoate (PFOA). J. Endocrinol. Investig..

[87] Temkin A.M., Hocevar B.A., Andrews D.Q., Naidenko O.V., Kamendulis L.M. (2020). Application of the key characteristics of carcinogens to per and polyfluoroalkyl substances. Int. J. Environ. Res. Public Health.

[88] Lu C., Cheng S. (2010). Thyroid hormone receptors regulate adipogenesis and carcinogenesis via crosstalk signaling with peroxisome proliferator-activated receptors. J. Mol. Endocrinol..

[89] Barry V., Winquist A., Steenland K. (2013). Perfluorooctanoic acid (PFOA) exposures and incident cancers among adults living near a chemical plant. Environ. Health Perspect..

[90] Vieira V.M., Hoffman K., Shin H., Weinberg J.M., Webster T.F., Fletcher T. (2013). Perfluorooctanoic acid exposure and cancer outcomes in a contaminated community: A geographic analysis. Environ. Health Perspect..

[91] Olsen G.W., Burlew M.M., Marshall J.C., Burris J.M., Mandel J.H. (2004). Analysis of episodes of care in a perfluorooctanesulfonyl fluoride production facility. J. Occup. Environ. Med..

[92] Leonard R.C., Kreckmann K.H., Sakr C.J., Symons J.M. (2008). Retrospective cohort mortality study of workers in a polymer production plant including a reference population of regional workers. Ann. Epidemiol..

[93] United States Environmental Protection Agency Basic Information about Pesticide Ingredients. https://www.epa.gov/ingredients-used-pesticide-products/basic-information-about-pesticide-ingredients.

[94] Centers for Disease Control and Prevention Reproductive Health and the Workplace. https://www.cdc.gov/niosh/topics/repro/pesticides.html.

[95] Aktar W., Sengupta D., Chowdhury A. (2009). Impact of pesticides use in agriculture: Their benefits and hazards. Interdiscip. Toxicol..

[96] Hurley P.M. (1998). Mode of carcinogenic action of pesticides inducing thyroid follicular cell tumors in rodents. Environ. Health Perspect..

[97] Schreinemachers D.M., Creason J.P., Garry V.F. (1999). Cancer mortality in agricultural regions of minnesota. Environ. Health Perspect..

[98] Zeng F., Lerro C., Lavoué J., Huang H., Siemiatycki J., Zhao N., Ma S., Dexiel N.C., Friesen M.C., Udelsman R. (2017). Occupational exposure to pesticides and other biocides and risk of thyroid cancer. Occup. Environ. Med..

[99] Jayaraj R., Megha P., Sreedev P. (2016). Organochlorine pesticides, their toxic effects on living organisms and their fate in the environment. Interdiscip. Toxicol..

[100] National Institute of Environmental Health Sciences DDT- A Brief Hsitory and Status. https://www.epa.gov/ingredients-used-pesticide-products/ddt-brief-history-and-status.

[101] Cohn B.A., Wolff M.S., Cirillo P.M., Sholtz R.I. (2007). DDT and breast cancer in young women: New data on the significance of age at exposure. Environ. Health Perspect..

[102] Goldner W.S., Sandler D.P., Yu F., Shostrom V., Hoppin J.A., Kamel F., LeVan T.D. (2013). Hypothyroidism and pesticide use among male private pesticide applicators in theagricultural health study. J. Occup. Environ. Med..

[103] Brucker-David F. (1998). Effects of environmental synthetic chemicals on thyroid function. Thyroid.

[104] National Institute of Environmental Health Sciences Hexachlorobenzene. https://www.epa.gov/sites/production/files/201609/documents/hexachlorobenzene.pdf.

[105] National Institute of Environmental Health Sciences Pentachlorophenyl. https://www.epa.gov/ingredients-used-pesticide-products/pentachlorophenol.

[106] Agricultural Health Study Questionnaires & Study Data. https://aghealth.nih.gov/collaboration/questionnaires.html.

[107] Lerro C.C., Freeman L.E.B., DellaValle C.T., Andreotti G., Hoffman J.N., Koutros S., Parks C.H., Shrestha S., Alavanja M.C.R. (2020). Pesticide exposure and incident thyroid cancer among male pesticide applicators in agricultural health study. Environ. Int..

[108] Louis L.M., Lerro C.C., Friesen M.C., Andreotti G., Koutros S., Sandler D.P., Blair A., Robson M.G., Beane Freeman L.E. (2017). A prospective study of cancer risk among agricultural health study farm spouses associated with personal use of organochlorine insecticides. Environ. Health.

[109] Saracci R., Kogevinas M., Bertazzi P.A., Bueno de Mesquita B.H., Coggon D., Green L.M., Kauppinen T., Abbé K.A., Littorin M., Lynge E. (1991). Cancer mortality in workers exposed to chlorophenoxy herbicides and chlorophenols. Lancet.

[110] Grimalt J.O., Sunyer J., Moreno V., Amaral O.C., Sala M., Rosell A., Anto J.M., Albaiges J. (1994). Risk excess of soft-tissue sarcoma and thyroid cancer in a community exposed to airborne organochlorinated compound mixtures with a high hexachlorobenzene content. Int. J. Cancer.

[111] Ruder A.M., Yiin J.H. (2011). Mortality of US pentachlorophenol production workers through 2005. Chemosphere.

[112] National Institute of Environmental Health Sciences Organophopshate Insecticides. https://www.epa.gov/sites/production/files/documents/rmpp_6thed_ch5_organophosphates.pdf.

[113] Centers for Disease Control and Prevention Organophosphorus Insecticides: Dialkyl Phosphate Metabolites. https://www.cdc.gov/biomonitoring/OPDPM_BiomonitoringSummary.html.

[114] Campos É., Freire C. (2016). Exposure to non-persistent pesticides and thyroid function: A systematic review of epidemiological evidence. Int. J. Hyg. Environ. Health.

[115] Centers for Disease Control and Prevention Toxic Substances Portal-Diazinon. https://www.atsdr.cdc.gov/ToxProfiles/tp.asp?id=512&tid=90.

[116] U.S. Food and Drug Administration Malathion. https://www.accessdata.fda.gov/drugsatfda_docs/label/2011/018613s017lbl.pdf.

[117] Mokarizadeh A., Faryabi M.R., Rezvanfar M.A., Abdollahi M. (2015). A comprehensive review of pesticides and the immune dysregulation: Mechanisms, evidence and consequences. Toxicol. Mech. Methods.

[118] Abdollahi M., Mostafalou S., Pournourmohammadi S., Shadnia S. (2004). Oxidative stress and cholinesterase inhibition in saliva and plasma of rats following subchronic exposure to malathion. Comp. Biochem. Physiol. C Toxicol. Pharmacol..

[119] Lerro C.C., Koutros S., Andreotti G., Friesen M.C., Alavanja M.C., Blair A., Hoppin J.A., Sandler D.P., Lubin J.H., Ma X. (2015). Organophosphate insecticide use and cancer incidence among spouses of pesticide applicators in the agricultural health study. Occup. Environ. Med..

[120] National Institute of Environmental Health Sciences Alachlor. https://www3.epa.gov/pesticides/chem_search/reg_actions/reregistration/fs_PC-090501_1-Dec-98.pdf.

[121] Wilson A.G., Thake D.C., Heydens W.E., Brewster D.W., Hotz K.J. (1996). Mode of action of thyroid tumor formation in the male Long–Evans rat administered high doses of alachlor. Fundam. Appl. Toxicol..

[122] Lee W.J., Hoppin J.A., Blair A., Lubin J.H., Dosemeci M., Sandler D.P., Alavanja M.C.R. (2004). Cancer incidence among pesticide applicators exposed to alachlor in the agricultural health study. Am. J. Epidemiol..

[123] Acquavella J.F., Delzell E., Cheng H., Lynch C.F., Johnson G. (2004). Mortality and cancer incidence among alachlor manufacturing workers 1968–1999. Occup. Environ. Med..

[124] EUR-Lex, Access to European Union Law. https://eurlex.europa.eu/eli/dec/2004/248/oj.

[125] Son H., Nishikawa A., Okazaki K., Lee K., Imazawa T., Hirose M. (2003). Lack of modifying effects of atrazine and/or tamoxifen on thyroid carcinogenesis in rats pretreated with N-bis (2-hydroxypropyl) nitrosamine (DHPN). Food Chem. Toxicol..

[126] Freeman L.E.B., Rusiecki J.A., Hoppin J.A., Lubin J.H., Koutros S., Andreotti G., Zahm S.H., Hines C.J., Coble J.B., Barone-Adesi F. (2011). Atrazine and cancer incidence among pesticide applicators in the agricultural health study (1994–2007). Environ. Health Perspect..

[127] Lerro C., Freeman L.B., Koutros S., Andreotti G., Hoffman J., DellaValle C., Alavanja M., Sandler D., Chen H., Blair A. (2016). O25-1 Pesticide use and thyroid cancer incidence among spouses of pesticide applicators in the agricultural health study. Occup. Environ. Med..

[128] Centers for Disease Control and Prevention (2017). Carbamate Insecticides Overview. https://www.cdc.gov/biomonitoring/Carbofuran_BiomonitoringSummary.html.

[129] National Institute of Environmental Health Sciences Organophosphathes (OP) and N-Methyl Carbamates. https://www.epa.gov/pesticide-reevaluation/groups-pesticides-registration-review#ops.

[130] Dias E., e Costa F.G., Morais S., de Lourdes Pereira M. (2015). A review on the assessment of the potential adverse health impacts of carbamate pesticides. Topics in Public Health.

[131] Dhouib I., Jallouli M., Annabi A., Marzouki S., Gharbi N., Elfazaa S., Lasram M.M. (2016). From immunotoxicity to carcinogenicity: The effects of carbamate pesticides on the immune system. Environ. Sci. Pollut. Res..

[132] Nordby K., Andersen A., Irgens L.M., Kristensen P. (2005). Indicators of mancozeb exposure in relation to thyroid cancer and neural tube defects in farmers’ families. Scand. J. Work Environ. Health.

[133] Boas M., Feldt-Rasmussen U., Main K.M. (2012). Thyroid effects of endocrine disrupting chemicals. Mol. Cell Endocrinol..

